# Considerations for the Safe Operation of Schools During the Coronavirus Pandemic

**DOI:** 10.3389/fpubh.2021.751451

**Published:** 2021-12-16

**Authors:** Ronan Lordan, Samantha Prior, Elizabeth Hennessy, Amruta Naik, Soumita Ghosh, Georgios K. Paschos, Carsten Skarke, Kayla Barekat, Taylor Hollingsworth, Sydney Juska, Liudmila L. Mazaleuskaya, Sarah Teegarden, Abigail L. Glascock, Sean Anderson, Hu Meng, Soon-Yew Tang, Aalim Weljie, Lisa Bottalico, Emanuela Ricciotti, Perla Cherfane, Antonijo Mrcela, Gregory Grant, Kristen Poole, Natalie Mayer, Michael Waring, Laura Adang, Julie Becker, Susanne Fries, Garret A. FitzGerald, Tilo Grosser

**Affiliations:** ^1^Institute for Translational Medicine and Therapeutics, Perelman School of Medicine, University of Pennsylvania, Philadelphia, PA, United States; ^2^Faculty of Science & Engineering, University of Limerick, Limerick, Ireland; ^3^Children's Hospital of Philadelphia, Philadelphia, PA, United States; ^4^Department of Microbiology, Perelman School of Medicine, University of Pennsylvania, Philadelphia, PA, United States; ^5^Department of Systems Pharmacology and Translational Therapeutics, Perelman School of Medicine, University of Pennsylvania, Philadelphia, PA, United States; ^6^Department of Genetics, Perelman School of Medicine, University of Pennsylvania, Philadelphia, PA, United States; ^7^Department of English, University of Delaware, Newark, DE, United States; ^8^Department of Civil, Architectural and Environmental Engineering, Drexel University, Philadelphia, PA, United States; ^9^Division of Neurology, Children's Hospital of Philadelphia, Philadelphia, PA, United States; ^10^Division of Public Health, University of the Sciences, Philadelphia, PA, United States

**Keywords:** COVID-19, SARS-CoV-2, education, vaccination, post-acute sequelae of SARS-CoV-2 infection (PASC), pediatric inflammatory multisystem syndrome temporally associated with SARS-CoV-2 (PIMS-TS), multisystem inflammatory syndrome in children (MIS-C)

## Abstract

During the severe acute respiratory syndrome coronavirus 2 (SARS-CoV-2) pandemic, providing safe in-person schooling has been a dynamic process balancing evolving community disease burden, scientific information, and local regulatory requirements with the mandate for education. Considerations include the health risks of SARS-CoV-2 infection and its post-acute sequelae, the impact of remote learning or periods of quarantine on education and well-being of children, and the contribution of schools to viral circulation in the community. The risk for infections that may occur within schools is related to the incidence of SARS-CoV-2 infections within the local community. Thus, persistent suppression of viral circulation in the community through effective public health measures including vaccination is critical to in-person schooling. Evidence suggests that the likelihood of transmission of SARS-CoV-2 within schools can be minimized if mitigation strategies are rationally combined. This article reviews evidence-based approaches and practices for the continual operation of in-person schooling.

## Introduction

The impact of the severe acute respiratory syndrome coronavirus 2 (SARS-CoV-2) pandemic on a generation of children can be anticipated to be extensive and long-lasting. In addition to the health consequences of coronavirus disease 2019 (COVID-19), the economic repercussions have brought financial insecurity to families and communities. Indeed, pre-existing social and health disparities has disproportionally affected many communities. School closures to reduce the number of person-to-person contacts and viral spread in the population (1) have caused prolonged interruptions to in-person learning and affected children's education and well-being. The benefit of formal education also is in the social, emotional, and behavioral dimensions that are difficult to replace in a distance learning environment. Therefore, enabling continuous in-person education is a central goal within the public health response to the pandemic.

Operating schools for in-person learning requires a dynamic approach as community spread fluctuates and more transmissible (and potentially more virulent) strains emerge (2–4). Key concerns include health risks to members of the school community and their families; disruption of learning, social, emotional and physical well-being associated with school closures, remote or hybrid learning, and quarantine periods; and the potential contribution of schools to sustaining viral spread in the population.

## Health Concerns Related to In-Person Learning

COVID-19 is predicted to evolve into an endemic disease in regions with infection and vaccination rates that confer inadequate protective population-level immunity (5, 6). Recurrent outbreaks among those who are not yet or are no longer immune are characteristic of many infectious diseases, including the common cold or influenza. Akin to their role in the infection dynamics of these seasonal respiratory infections, schools may become reservoirs of SARS-CoV-2 due to their dense social setting if sufficient protective measures are not taken to limit the propagation of natural infections among susceptible individuals (7). Vaccination against SARS-CoV-2 markedly lessens, if not eliminates, most health concerns associated with in-person schooling for adults and children who are immunized—even in regions where highly transmissible viral variants predominate (8–14). However, those in a school community who are not yet eligible for vaccination, who have not had an opportunity to be vaccinated, or who choose to delay or forgo vaccination remain at risk.

The susceptibility of unvaccinated school-aged children to SARS-CoV-2 infection and this population's likelihood of spreading the virus has been widely discussed since lower rates of reported infections in children than adults were noted (15). However, the cause of this age-based disparity is likely multifactorial. Hypotheses include decreased exposure (16), missed asymptomatic cases (17), and a lower biological susceptibility to being infected (15, 18, 19). The interplay of these factors may contribute to heterogeneity in reports on pediatric susceptibility to SARS-CoV-2 infection. While some contact tracing studies estimated a reduced infection risk of children and adolescents (15, 20–24), others found no difference when compared with adults (25, 26). Similarly, although some seroprevalence studies found SARS-CoV-2 antibodies as a marker of past infection in fewer children than adults, others observed no or only small effects of age (27). Additionally, evidence of potential differences in the risk of SARS-CoV-2 infection between younger and older school-aged children are not uniformly supported and may relate more to study design than age-dependent biology (15, 20–26). For example, a large surveillance testing study in Austria found no differences in the infection rates among 6–10 and 11–14 year olds, suggesting that primary and middle school students have similar risks of infection (28).

There is more clarity regarding children's ability to spread the virus. Minor age-based differences between nasopharyngeal viral loads exist, but they do not suggest that children are substantially less infectious than adults (29). Indeed, data from India provide evidence that children and adolescents of all ages are effective transmitters of the disease (30). This is supported by reports of increased prevalence of SARS-CoV-2 in children, particularly among adolescents. For example, during the second pandemic wave in the UK (November 2020), the highest prevalence of SARS-CoV-2 positive tests was obtained from adolescents who attended school (31). This was in the context of the more transmissible virus variant Alpha (B.1.1.7) (31). When the highly transmissible Delta (B.1.617.2) variant began to spread in the British population (June 2021), this age group again showed the highest infection rate in the population (32) and it appeared that some case clusters were associated with schools (4). Similarly, a rapid increase of infections and hospitalizations in children and adolescents was observed in the U.S. when Delta began to spread in July 2021 (14, 33). By October 2021 ~25% of newly reported COVID-19 infections occurred in children under the age of 18 years, an age group that makes up 22 % of the population in the U.S. (34). These findings are in accordance with a contact tracing study that saw the highest probability of transmission among students aged 10–19 years (35). Because of our evolving understanding of how susceptible to infection with increasingly transmissible virus variants children are and how they contribute to the spread of SARS-CoV-2, schools should not rely on an assumed natural protection of (and by) children. Mitigation plans for all age groups continue to be necessary to curb the spread of virus variants in schools.

While the age dependency of infection risk is controversial, there is strong evidence that children who are infected with SARS-CoV-2 have a substantially lower risk of severe outcomes than adults. Most are diagnosed with asymptomatic or minimally symptomatic infections (36–41) or experience a mild form of respiratory disease that will typically resolve within a week or two (25, 42–46). Increasing evidence suggests that the airway epithelium of children may have a better ability to sense infection with SARS-CoV-2 and may mount a more pronounced immune response than adults, resulting in faster viral clearance (47–49). However, even in mild cases, individuals can experience persistent symptoms for weeks following a SARS-CoV-2 infection. These prolonged post-acute sequelae of SARS-CoV-2 infection, referred to as “PASC” or “long-COVID,” have only begun to be studied in children. As in adults, symptoms include fatigue, dyspnea, chest pain, cardiac palpitations, myalgia, gastrointestinal issues, headache, anosmia, and issues with memory and concentration (50, 51). Preliminary studies suggest that the most common persistent symptoms in children are fatigue and cough, although longstanding insomnia, headache, and pain were also frequently reported (52–54). One study found that ~40 % of children diagnosed with COVID-19 reported at least one persistent symptom 2 months post infection (55). Another study estimated that 10% of children may still experience symptoms 3 months post infection (56). In a large, matched cohort study, the incidence rate of health problems was ~30% higher in the COVID-19 cohort than in matched controls at least 3 months after infection (57). Symptoms including fatigue, cough, throat or chest pain, headache, fever, and abdominal pain occurred 1.45 to 2.28 times more frequently in the COVID-19 than in the control cohort. Another study that focused on severely ill children that were hospitalized with COVID-19 estimated that 25% may experience persistent symptoms 5 months post discharge with higher risk in those with pre-existing allergic diseases. Some symptoms persisted even longer (58). The impact of this persistent symptomatology on academic participation and performance is still to be determined.

Despite its overall rarity, children can become severely ill with COVID-19 (59, 60) and just as in adults, racial, ethnic, and socioeconomic disparity in disease severity is evident (25, 61). Exact rates of severe disease are difficult to estimate because of the large number of undetected infections and prophylactic admission to hospitals, which result in an overestimate in unadjusted health statistics of COVID-19 severity (17). Considering these limitations, a reported cumulative (October 2021) hospitalization rate of 0.8% of all laboratory confirmed infections in the pediatric U.S. population represents a conservative estimate of the rate of severe disease (62). Notably, over one quarter had a pre-existing condition such as obesity, type I diabetes, asthma, and neurodevelopmental disorders (63). Youths (12–17 years) and young children (0–4 years) were ~2–3 times more frequently hospitalized than 5–11 year old children (64). Approximately one in four children admitted to a hospital in the U.S. received intensive care (14, 65). In Germany where the hospitalization rate of 1–17 year olds with a documented SARS-CoV-2 infection has also been ~1%, it was determined that between 1% and 10% of children admitted to a hospital received intensive care (66).

A rare, severe, delayed consequence of COVID-19 is multisystem inflammatory syndrome in children (MIS-C), also referred to as pediatric inflammatory multisystem syndrome temporally associated with SARS-CoV-2 (PIMS-TS) (67–73). MIS-C is characterized by persistent fever and systemic inflammation, involving at least two organs (i.e., lungs, heart, kidneys, skin, blood, gastrointestinal tract, and brain), weeks after infection with SARS-CoV-2. As of November, 2021, the Centers for Disease Control and Prevention (CDC) report a median age of 9 years (interquartile range 5–13 years) for MIS-C in the U.S. (74). Initial estimates suggest an MIS-C incidence of ~2 per 100,000 children and adolescents in the U.S. population during the first year of the pandemic (68) before the number of infected children rose steeply when the Delta variant began to spread. During the same period (pre Delta) ~3,500 COVID-19 infections per 100,000 children and adolescents were laboratory confirmed (34). Because the number of total infections including undetected infections can be expected to be markedly higher (17), a rough estimate might be that between 1 in 1,000 and 1 in 10,000 infected children and adolescents have developed MIS-C in the U.S.. An analysis of MIS-C reports in seven U.S. states during the first months of the pandemic estimated that ~3 in 10,000 infected children developed MIS-C (75, 76). German registry data found rates of 7 in 1,000 and 4 in 10,000 for 1–10 and 12–17 year old children and youths with documented infections suggesting that the case rate is similar in both regions (66). Emerging data indicates that while many children with MIS-C require intensive care, most recover fully or with minimal residual functional impairment (70). However, as of November, 2021, the CDC's national registry lists 46 deaths among 5,217 patients (~0.9 %) meeting the MIS-C case definition (75).

An infection fatality rate (IFR) can be estimated as a risk for death among infected individuals. This value accounts for both laboratory confirmed and imputed undetected infections. The IFR of the H1N1 pandemic 2009 influenza is estimated to be <1 in 100,000 infected school-aged children (77, 78), and the fatality rate of measles may approximate 1–2 in 1,000 infected school-aged children (79). In school-aged children with SARS-CoV-2, an IFR of 1–3 in 100,000 has been estimated (80, 81). The IFR may be higher in children under the age of 4 years (80, 81). In comparison, the estimated IFR for adults aged 35 to 44 years ranges from 40 to 75 in 100,000 infected persons and the IFR for adults aged 65 to 74 years ranges from 1,075 to 1,670 in 100,000 infected persons (80).

At the population level, COVID-19 ranked among the 10 leading causes of death among U.S. children throughout most of 2021 (82). A mortality rate of ~0.2 in 100,000 children has been attributed to COVID-19 during the first year of the pandemic (83, 84). Children aged 3–10 had the lowest COVID-19 related mortality (85). Notably, the COVID-19 related mortality was about two-fold higher than the typical mortality rate of influenza in school-aged children in prior years (range 0.10–0.13 in 100,000) (85, 86). However, comparison of COVID-19 related mortality with historical influenza data is complicated, because COVID-19 deaths occurred despite unprecedented protective public health measures. There were 144 to 198 annual pediatric influenza deaths in the U.S. in the 3 years prior to the 2020/21 season (October to May), during which only one influenza-related pediatric death occurred (87). This suggests that the public health measures instituted, including school closures and hybrid learning, may have had broad impact on reducing the transmission of other viruses. In contrast to the isolated pediatric death from influenza in the 2020/21 season, over 220 pediatric COVID-19 deaths were reported in the U.S. during the same time (34, 62). These observations highlight inherent differences in infection dynamics between these two diseases.

As public health mitigation measures are lifted in increasingly vaccinated populations, it remains unclear how this will impact COVID-19 related morbidity and mortality in school age children. Additionally, there is an unmet need to understand the frequency and severity of PASC in children. The emergence of more transmissible (and potentially more virulent) variants may also affect disease burden in the pediatric population. Moreover, the ethical considerations regarding phasing out protective measures for children who are age-eligible but remain unvaccinated are different from those in the adult population. Because of these evolving uncertainties, protecting students should remain a priority, even when many adults and age-eligible students have been immunized.

## Impact of School Closures, Remote or Hybrid Learning, and Quarantine Periods

Since the beginning of the pandemic, education has rotated between remote and in-person learning. However, even when in-person learning was offered quarantine and isolation periods for COVID-19 infections and exposures affected as many as 20% of students during pandemic waves in both Spain and China (3, 88, 89). Shifts between modes of learning have led to negative social, emotional, educational, and physical consequences not only through reduced physical presence in school buildings, but also through loss of essential services for children provided by schools (90–92). Many receive their main source of nutrition in schools. For others school is a place of refuge and protection. For example, child maltreatment was underreported by an estimated 27% in the U.S. during the pandemic, likely because it went unnoticed or unreported due to lack of face-to-face interactions with teachers or other school personnel during school closures and remote programs (93). Many schools were unable to provide mental and behavioral health services to students while working remotely, which may have contributed to under recognition of child maltreatment and more generally increases in stress and anxiety (94, 95).

The impact of the extended school closures during the pandemic remains to be fully appreciated, but students in the Netherlands showed little progress while learning from home during the first lockdown (96). In the U.S., it was projected that students would have ~40–50% of the learning gains in mathematics and 60–70% of the learning gains in reading relative to a typical school year, although the top third of students may have been impacted less (97). Learning loss was reported to be more prevalent for students of color and for socioeconomically disadvantaged students (96, 98). Moreover, students with disabilities were less likely to have had access to educational support (99).

Despite being a necessary public health response to the pandemic, even limited quarantine measures and temporary school closures in response to infections and outbreaks in the school community can have negative consequences— perhaps most for children of working parents. Remote learning may disadvantage students further if the household is not digitally competent (88). Academic outcomes are thought to be similarly affected by transitions to distance learning as they are by absenteeism (100). Home confinement for just 8–10 days can measurably impair emotion regulation and increase symptoms of anxiety and depression (101). Increased screen time can negatively affect sleep patterns and limit exercise, which impacts both physical and mental health (102). Perhaps most fundamentally, interruptions to daily in-person interactions with classmates, and a network of caring adults may potentially cause damaging isolation-related effects such as anxiety and depression (101, 103). Disadvantaged children, such as refugees, migrants, and those with cognitive and/or physical disabilities are likely to have faced greater exclusion from learning (104). Therefore, minimizing disruption of continuous in-person learning by encouraging vaccination and implementing proactive infection control measures that identify potentially infected individuals early and limit the likelihood of spread is a central goal of school operation plans.

## Schools and Community Spread

At its peak, COVID-19-related school closures, which were based on the response plans to influenza pandemics, affected an estimated 1.5 billion or 90% of the world's learners (105, 106). Estimating the contribution of specific interventions to overall mitigation during countrywide general lockdowns is challenging. However, retrospective modeling analyses suggest that schools may have played an important role in disseminating infections into the population during the initial wave of SARS-CoV-2 before mitigation measures were implemented (1, 16, 107). Indeed, large viral outbreaks among students and teachers, which spread to family members and other close contacts outside of school, have been documented when preventive measures were insufficient or were insufficiently followed (38, 108–112).

Early in the pandemic, in person schooling was associated with increased rates of infection (113). In Sweden, family members of students and teachers who were physically present in school buildings had higher rates of infections than family members of students and teachers who engaged in remote learning (113). As our experiences evolved, it was found that the risk for in-school transmission correlated with community exposure risk (109, 114–121). National and regional contact tracing and surveillance data collected in Europe, Australia, and Singapore during periods of low viral circulation (summer/fall 2020) found that clusters of cases or documented outbreaks were infrequent despite variable degrees of mitigation measures (109, 114–121). Most infections appeared to have been acquired outside of school. Employees were found to be at a higher risk of seeding SARS-CoV-2 infections than children in U.K. schools (114). Random COVID-19 testing among Austrian school students and staff showed that the number of infections detected in schools increased with the surging incidence of infections in the broader community (28). Similarly, widened viral circulation in a region coincided with a growing number of outbreaks in Australian schools (122). Conversely, an increasing vaccination rate in the adult population was associated with a substantial decrease in cases in children and teenagers in Israel before they became age-eligible for vaccination (123). This direct relationship between the infection dynamics in schools and the surrounding population highlights the importance for safe school operation of reducing the infections in the community.

The question of how much in-person learning contributed to the dissemination of infections into the regional population is difficult to address. Two analyses of German incidence data found that SARS-CoV-2 transmission in the population decreased following school reopening after the summer break 2020 under conditions of low baseline viral circulation. This was hypothesized to relate to reduced travel as the summer break ended and more widespread adoption of infection mitigation behaviors by the population (124, 125). In Michigan and Washington, a temporal association between re-opening schools (fall 2020) and infections in the broader population was observed only in regions with high baseline infection activity (126). Where incidence rates were low prior to the begin of in-person learning, no increase in viral transmission in the population was observed (126, 127). Similar observations were made in schools in Sicily (128). Moreover, a nationwide analysis showed no evidence of increased hospitalizations for COVD-19 in U.S. regions with low baseline disease activity where schools re-opened for hybrid or full in-person learning (129). However, an impact of in-person schooling could not be excluded in counties with high baseline disease activity (129). Indeed, a more recent nationwide analysis of U.S. counties found an overall increase of new COVID-19 cases and deaths in communities in temporal association with school opening from April to December, 2020 (130). In counties where teaching resumed in-person but without a mask mandate in schools, the rise in case numbers and deaths was steeper and more pronounced than in counties where masks were required (130).

**Figure 1:**
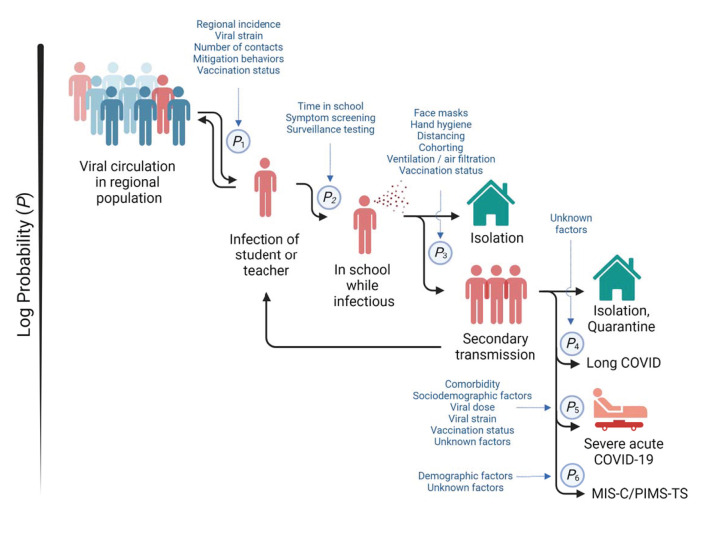
Infection dynamics in schools. Multiple factors and mitigation measures (in blue) modify the probabilities of (*P*_1_) infection of students or teachers outside of school, (*P*_2_) the import of infection into school, (*P*_3_) the spread among students and teachers, (*P*_4_) development of prolonged post-acute sequelae of SARS-CoV-2 infection (“long COVID”), (*P*_5_) progression to severe acute COVID-19, and (*P*_6_) development of multisystem inflammatory syndrome in children (MIS-C), also referred to as pediatric inflammatory multisystem syndrome temporally associated with SARS-CoV-2 (PIMS-TS). Viral circulation in the regional population is a key determinant of infection risk of students and teachers. Secondary transmission within schools can amplify infections in the regional population.

Overall, these data suggest that the infection dynamics in schools and in the surrounding population are in dynamic equilibrium (Figure 1). This illustrates the importance of continued suppression of viral transmission in the broader population while the vaccination rate increases through effective public health measures such as mask wearing and limits on social gatherings. Adults play a particular role in the infection of children with ~70% of pediatric cases being secondary to an adult case (131). However, young children are not yet age-eligible for vaccination in many regions in the world, or supply shortages limit availability of the SARS-CoV-2 vaccines for those who are eligible. Therefore, the interplay between community spread and outbreaks in schools supports a strategy of prioritizing older adults for vaccination where supply is limited, not only because they are most at risk but because mitigating their role in community spread can also be expected to render in-person learning safer (123). However, this still requires mitigation of transmission within schools to minimize the risk of larger outbreaks. Adults bare the greater responsibility to monitor their actions within the community to prevent the spread of SARS-CoV-2 among children (132).

Emerging evidence suggests that mitigation measures in schools are effective in maintaining low levels of SARS-CoV-2 transmission among students and teachers (secondary transmission) (133, 134). A study in primary schools, nurseries, and kindergartens in Germany determined that asymptomatic spread among these age groups was low when mitigation measures including mask wearing, and classroom ventilation were appropriately applied (135). An Australian study in schools that practiced hand hygiene, physical distancing, reduced mixing of students and reduced after-school activities, but not mask wearing or enhanced classroom ventilation or filtration, found 1 secondary infection case per 9 infectious students who attended school (15). A German analysis of contact tracing data in schools with a 50% reduction of classroom occupancy through hybrid learning, regular room ventilation, limited contacts between cohorts, exclusion with minimal symptoms, but no mask wearing and no distancing in classrooms estimated a frequency of 1 secondary case per 12 infectious students who attended school (136). A report on Norwegian schools that used similar mitigation measures but no masking in classrooms, found no secondary cases among 13 students who attended school while considered infectious (120). A study conducted in Wisconsin observed 1 secondary case per 20 infectious students in schools that consistently practiced mask wearing, had cohort sizes of ≤ 20 students, limited contacts between cohorts, but had no symptom screening program (137). Similarly, school districts in North Carolina detected 1 secondary infection per 24 community-acquired infections in schools that practiced mask wearing, physical distancing, hand hygiene, and reduced cohort mixing (138). Collectively, these studies which were conducted before the Delta variant became dominant, indicate that instituting basic mitigation strategies can enable in-person schooling with a low risk of secondary SARS-CoV-2 transmission. However, the impact of more transmissible virus variants such as Delta remains less understood and can be expected to require additional preventive measures.

## Synergistic Risk Mitigation in Schools

Presently, no single intervention, including vaccination, completely prevents viral transmission. However, combined mitigation strategies may be synergistically beneficial (133, 139–141). In safety research this concept of risk management is sometimes described as the “Swiss Cheese Model”, where multiple defensive layers are combined with the understanding that each individual layer has holes. The combination of multiple layers provides safety through redundancy (142). Evidence based choices need to be made as to which mitigation strategies to combine to adapt to changing risk levels and maximize learning.

### Reducing the Risk of Infections Being Brought Into the School

Given the association between cases in schools with burden of disease in the community, the most effective approach to enable safe in-person learning is to focus on broader public health measures that suppress viral spread in the population (Figure 1). The SARS-CoV-2 vaccines are effective in preventing severe disease and reduce the likelihood of infection and transmission of the virus (123, 143). As such, widespread vaccination of the eligible population is a potent tool in reducing viral spread in the unvaccinated, younger population. Household members are the primary source of infection of school-aged children, and extracurricular activities are a source of infection among adolescents (131, 140, 144). Thus, the entire community of those who interact with unvaccinated children can contribute significantly to protecting schools by getting vaccinated.

Daily symptom-based surveillance combined with aggressive diagnostic testing for COVID-19 infections is effective in reducing the likelihood of having infectious students in schools (140, 145). As many symptomatic students who are infected with SARS-CoV-2 present with mild symptoms, such as rhinorrhea, cough, sore throat, or headache (146), there should be a low threshold for diagnostic COVID-19 testing. Additionally, daily health screening affords the opportunity to gain insights into the overall risk profile of the larger school community including household members by asking about potential contacts of students with symptomatic or laboratory confirmed infected persons.

Silent transmission by individuals who have not yet developed symptoms (“presymptomatic”) or who will never notice symptoms (“persistently asymptomatic”) contribute to the difficulty in containing the pandemic. Surveillance testing approaches in schools are used for identifying pre- / asymptomatic persons who are carrying the virus (147). Most reliable and sensitive diagnostic tests detect the SARS-CoV-2 genome using reverse transcriptase polymerase chain reaction (RT-PCR) (148, 149). Comparative studies suggest that saliva based diagnostic testing accuracy may be similar to that of nasopharyngeal swabs (150). However, slow turn-around times to results of tests based on nucleic acid amplification may not allow for timely isolation of an infected person who has no symptoms. Rapid antigen tests detect viral proteins in a sample of either saliva or a swab of the nose or throat using a lateral flow method similar to pregnancy tests. These tests provide an opportunity to identify pre- or asymptomatic carriers in real-time (151). Although they are generally less sensitive than RT-PCR tests, rapid antigen tests are effective for detecting individuals with high viral loads who would be considered presently infectious (149, 152–154). Saliva-based rapid tests are easy to perform at home or in school, but their sensitivity appears to be substantially lower than those that use nasal swabs (155, 156). Concerns related to surveillance testing include both false negative (the test is reported as negative, but the virus is present) and false positive (the test is reported as positive, but the virus is not present) results (157). The consequences of a false positive test (i.e., unnecessary isolation) can be limited by confirmatory testing using RT-PCR. Regular, for example weekly or more frequent testing reduces the impact of false negative test results (158, 159). Importantly, given the time course of viral load in throat and nose, the sensitivity of the test is less important for effective surveillance testing than the frequency of testing and turnaround time of result reporting (158).

While limited studies have systematically examined surveillance testing in schools, even a testing frequency as low as every 2 weeks appears to provide some balance to the false negative vs. false positive detection rate among students (160, 161). However, because a school wide testing-based surveillance system can identify some, but not all pre- / asymptomatic individuals, even frequent testing should not be relied on as the only strategy to enable in-person schooling. Additionally, school wide testing-based surveillance is not feasible everywhere considering the complex logistics and the costs, particularly in less affluent regions. Despite these hurdles, nationwide surveillance testing for schools has been successfully implemented in many countries, (162, 163), and is being planned or piloted in others (164–166).

An approach to reducing disruptions of learning when COVID-19 positive individuals have been identified in schools is to quarantine only close unvaccinated contacts, i.e., immediate classroom neighbors of students presumed infectious, instead of quarantining all of the unvaccinated students in a learning cohort. Typically, this is combined with proactive COVID-19 testing among the cohort. Another approach is to allow even close unvaccinated contacts of a student presumed infectious to remain in the classroom but require them to test frequently (“test-to-stay”). Currently, there is little empirical data in support of either approach. However, computational simulation suggests that only quarantining close contacts of an infected student rather than the entire class cohort, would not be predicted to increase the probability of large outbreaks in a school as long as SARS-CoV-2 infections in the school community are infrequent and adequate mitigation procedures are in place (i.e., face masks, ventilation, distancing where possible) (161, 167).

In addition to limiting individual health risks by decreasing the likelihood of illness, hospitalization, and death (8–14), COVID-19 vaccines provide a potent additional layer of protection for the school community by reducing the likelihood of infection. For example, the mRNA BNT162b2 COVID-19 vaccine (Pfizer-BioNTech, Comirnaty) has been estimated to reduce the risk of infection with the Delta variant by approximatively four-fold in adults and 10-fold in adolescents (13, 168). Although breakthrough infections can occur in vaccinated individuals (169), emerging evidence suggests that their infectiousness even with the Delta variant might be reduced (170). However, this effect declines with time and so a third (booster) vaccine dose may be needed. A recent study performed in Sweden observed that immunization extended protection from infection to family members who are not vaccinated (171). For example, 2 immunized family members reduced the infection risk for the non-immune family members by 75 to 86%, whereas 3 immunized family members reduced the infection risk for the non-immunized family members by 91% to 94%. Therefore, it can be expected that the likelihood of COVID-19 outbreaks will decline substantially in increasingly vaccinated school communities (i.e., teachers, staff, students, families).

### Reducing the Risk of Transmission Within Schools

SARS-CoV-2 is spread through liquid particles that are generated by expiratory activities such as breathing, speaking, coughing, and sneezing (172). Liquid particles vary in size by orders of magnitude, ranging from larger droplets to smaller particles dispersed as aerosols. Large droplets (e.g., >50–100 μm) can settle quickly out of the air onto surfaces, so that individuals who are farther away from the droplet source are less likely to inhale them. Viral transmission by large exhaled droplets and secreted respiratory fluids is the basis for physical distancing and hand hygiene recommendations. While 1.8 m (6 foot) separation in classrooms was recommended for U.S. school reopening in fall 2020 and 1.5 m (5 foot) for European schools, more recent observational data suggest that a 0.9 m (3 foot) separation may be sufficient when students are seated and wear facemasks in classrooms (173). The impact of more transmissible variants on distancing requirements remains to be determined. Incorporating instruction on handwashing into the curriculum generally reduces the transmission of infectious diseases in schools (174). Antibacterial adjuvants to soap do not have greater efficacy at preventing infection and may be associated with antibiotic resistance (175). Hand sanitizers are an alternative, but they are not considered as effective as hand washing (176) and are an ingestion risk (177).

Unlike droplets, aerosols (e.g., 10^−4^ to 10 μm**)** can stay airborne for hours or until the air is exchanged (178). The risk for virus transmission while outdoors is much lower than indoors because moving air disperses and dilutes aerosol concentrations quickly (179). An important component of mitigation measures targeting indoor air quality is to reduce the release of aerosols by wearing facemasks and by limiting activities indoors that produce high concentrations of aerosols, such as shouting, singing, gym exercise, playing of wind instruments, or having large numbers of students in a classroom (180–183). Well-fitted face masks worn over mouth and nose effectively reduce viral transmission by reducing both emission and inhalation of viral particles (184, 185). Guidance surrounding what age groups should be wearing masks varies (186), but in the U.S., masking is generally recommended for children over 2 years old (187, 188) whereas the World Health Organization (WHO) recommends masks for children over 5 years old (189). A recent study in France found that while many mild inconveniences were reported by parents of children wearing masks, overall, children tend to tolerate them well (190). While wearing face masks does not impair the respiratory function of children (186), even in those as young as 2 years old (191), they reduce the capability to recognize emotional expressions and some mask types can impair speech intelligibility (192–194). Adults interacting with children should be aware of these limitations and compensate where possible. In how far the impact of facemasks on visual and auditory cues may affect language development in young children has not yet been systematically examined.

Additionally, ventilation and filtration strategies can remove virus particles from the air. The effectiveness of ventilation by opening windows or expelling indoor air with fans can be monitored by measuring CO_2_ levels as a proxy of the cumulative expiratory activity by the occupants (195), which has been a strategy considered by school authorities in Ireland (196). To supplement ventilation, or when it is not practical, air filtration systems should be used. Coronaviruses are found associated with water vapors, proteins, and salts in aerosolized particles often >1.0 μm, which can be effectively removed by filtration (19). Filters for heating, ventilation, and air conditioning (HVAC) systems with a minimum efficiency reporting value (MERV) of 13 have been suggested as a potential air purifying strategy in schools (197, 198). Standalone portable air cleaners that have high efficiency particulate air (HEPA) filters, which are capable of filtering 0.3 μm particles at 99.97% efficiency can supplement or replace centrally located filters (199). Schools that implemented measures to improve ventilation by keeping windows and doors open or using fans had a 35% lower incidence and schools that combined ventilation with filtration had a 48% lower incidence than schools that did not focus on indoor air quality (133).

For most respiratory viruses, the risk of infection depends on the number of viral particles a person inhales, or the viral inhaled dose, which is a function of breathing rate, inhaled concentration, and time of exposure (200). The dose of viral exposure may be proportional to the severity COVID-19 (201–204). It is plausible that prevention of transmission in schools is due to multiple layers of mitigation synergistically minimizing the viral dose students and teachers are exposed to rather than by eliminating exposure entirely.

### Limiting the Size of Potential School Outbreaks

Many of the infections in a population are spread by a disproportionately small number of people (205). Studies estimate that only 10–20% of coronavirus patients are responsible for 80% of all new infections of the early virus strains (206, 207). While more transmissible strains may spread more widely, outbreaks of viruses with such a hyperdispersed distribution are often driven by super-spreading events. Many super-spreading events involve crowded indoor places, loud speaking or singing, and inadequate use of protective measures such as face masks and physical distancing. The distribution of the sizes of reported outbreaks in schools reflects such a hyperdispersed distribution. Often, no or few new secondary infections are observed following exposures in school, while larger or very larger outbreaks are less frequent (167, 208). Banning larger group gatherings is a practice that avoids super-spreading and is highly effective in suppressing viral spread (1). Avoiding student mixing by creating protective (classroom) cohorts may allow clusters of infection to be more easily contained through limited quarantine measures. However, while this is feasible in early childhood, elementary and middle schools, a typical high school rotation structure creates much more complex networks of person-to-person contacts, which are predicted to result in a higher risk of outbreaks (161). Indeed, a study in Catalonia which was conducted in schools that practiced cohorting found that the secondary attack rates were higher in high school students than in preschool students (208). However, even in situations where protective cohorts are difficult to implement, a focus on limiting longer and closer interactions to members of the same group of individuals whenever feasible may still provide some benefit (209).

Alternating (“hybrid”) in-school and remote-learning models reduce the size of the protective cohort. Computational modeling suggests that weekly rotations (i.e., 5 days in school, 5 days remote) or groups of students rotating 2–3 days in school every week limit the number of contacts between students and teachers to reduce the likelihood of transmission events in schools (161, 210). Existing out-of-school social networks among students may guide such cohort formation and lead to a more effective reduction of contacts (211). However, whether these predictions translate into measurable effects on secondary cases in schools remains to be tested. At the population level, no difference in the incidence rates between hybrid and full in-person learning were apparent in Michigan (126). Therefore, limiting the educational benefit of full in-person instruction by implementing hybrid models should be weighed carefully against mitigating measures such as mask wearing, maximizing distance between individuals and room ventilation / air filtration. Since reduction of in-school time may disadvantage younger students disproportionately, this mitigation tool should be reserved primarily for older middle and high school students.

## Concluding Remarks

Societies have struggled worldwide to balance the obligation to provide quality education with minimizing the infection risk. However, the role of the broader community in preventing infections in schools has been a largely neglected consideration. To protect the school communities, there must be a collective acceptance for continuing general public health measures such as wearing masks in dense social settings, limiting contacts, activities with elevated risks of viral spread, and to avail of vaccination and testing. While the risk of severe illness and death related to COVID-19 are low in school-aged children, it is not negligible, and marked uncertainties regarding the long-term sequelae exist. The role of school in community spread and the negative impact of disruptions to in-person schooling by quarantine measures remain strong arguments to focus on protecting schools. Children deserve safe access to education. To achieve this, schools need a dynamic mitigation plan (Figure 1), community investment in public health measures, and the systematic collection of data to assess which strategies are most effective.

## Author Contributions

RL and TG: conceptualization, visualization, investigation, writing—original draft, and writing—review & editing. SP: conceptualization, investigation, writing—original draft, and writing—review & editing. EH, AN, SG, GP, CS, KB, TH, SJ, LM, ST, AG, SA, HM, S-YT, AW, LB, ER, PC, AM, and GG: investigation and writing—original draft. KP and NM: writing and reviewing & editing. MW: investigation and writing—review & editing. SF: conceptualization, investigation, and writing—review & editing. GF: conceptualization, writing—review & editing, and funding acquisition. All authors contributed to the article and approved the submitted version.

## Funding

This work was supported by a Grant (UL1TR001878) from the National Center for Advancing Translational Science. Dr. Bottalico was supported by National Institutes of Health Grants K12GM081259 University of Pennsylvania Postdoctoral Opportunities in Research and Teaching and T32ES01985 Translational Research Training Program in Environmental Health Sciences. Dr. Skarke is the McNeill Fellow of Translational Medicine and Therapeutics. Dr. FitzGerald is the McNeil Professor of Translational Medicine and Therapeutics.

## Conflict of Interest

LA serves on the medical advisory board of the MLD Foundation, CureMLD and “Don't Forget Morgan”. She is a consultant for Orchard Therapeutics, Biogen, and Takeda. She receives compensation and/or research funding for these roles. Additionally, she serves as an uncompensated member of the Board of Trustees and the scientific COVID-19 advisory committee of the Waldorf School of Philadelphia. GF is a chief scientific advisor for the journal Science Translational Medicine and is a senior advisor to Calico Laboratories and receives compensation for both roles. TG serves as an editor for the journal Circulation Genomic and Precision and receives compensation from the American Heart Association for this work. He was an uncompensated member of the Board of Trustees of the Waldorf School of Philadelphia and serves on the school's scientific COVID-19 advisory committee without compensation. The remaining authors declare that the research was conducted in the absence of any commercial or financial relationships that could be construed as a potential conflict of interest.

## Publisher's Note

All claims expressed in this article are solely those of the authors and do not necessarily represent those of their affiliated organizations, or those of the publisher, the editors and the reviewers. Any product that may be evaluated in this article, or claim that may be made by its manufacturer, is not guaranteed or endorsed by the publisher.
